# P-1803. Evaluation of Ganciclovir Dosing Strategies for the Treatment of Cytomegalovirus

**DOI:** 10.1093/ofid/ofaf695.1972

**Published:** 2026-01-11

**Authors:** Caroline Rosario, Stephanie Welch, Kiran Gajurel, Rupal Jaffa

**Affiliations:** Roper St. Francis Healthcare, Charleston, SC; Advocate Health: Atrium Health Antimicrobial Support Network, Charlotte, North Carolina; Atrium Health (Advocate Health), Charlotte, North Carolina; Advocate Health: Atrium Health Antimicrobial Support Network, Charlotte, North Carolina

## Abstract

**Background:**

Ganciclovir (GCV) exhibits high interindividual variability in pharmacokinetic models. A combination of the inherent complexity of patients with cytomegalovirus (CMV) and the adverse effect profile of GCV has led to variable dosing practices. The purpose of this study was to assess GCV dosing practices for treatment of CMV and associated clinical outcomes across a large health system.

**Figure 1:**
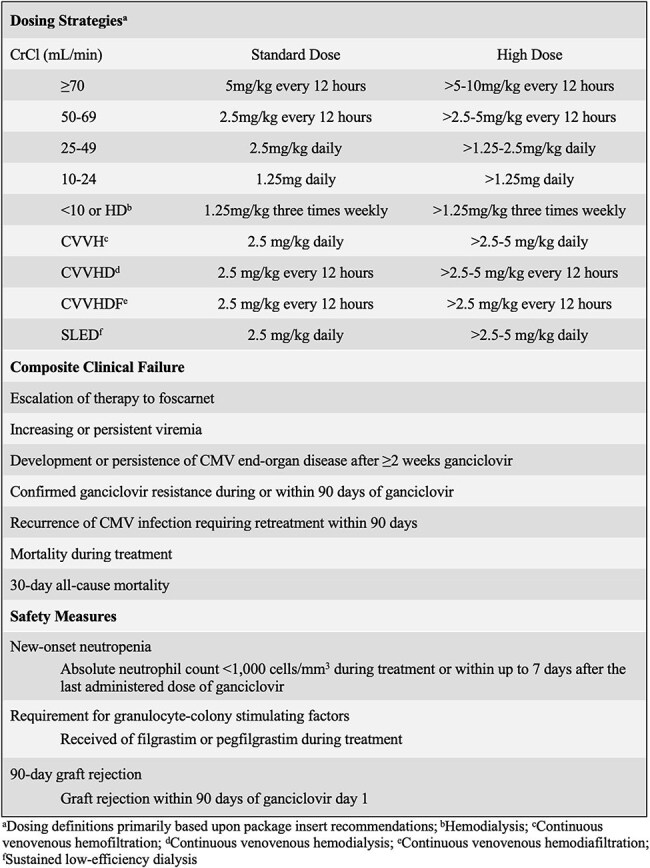
Definitions

**Figure 2:**
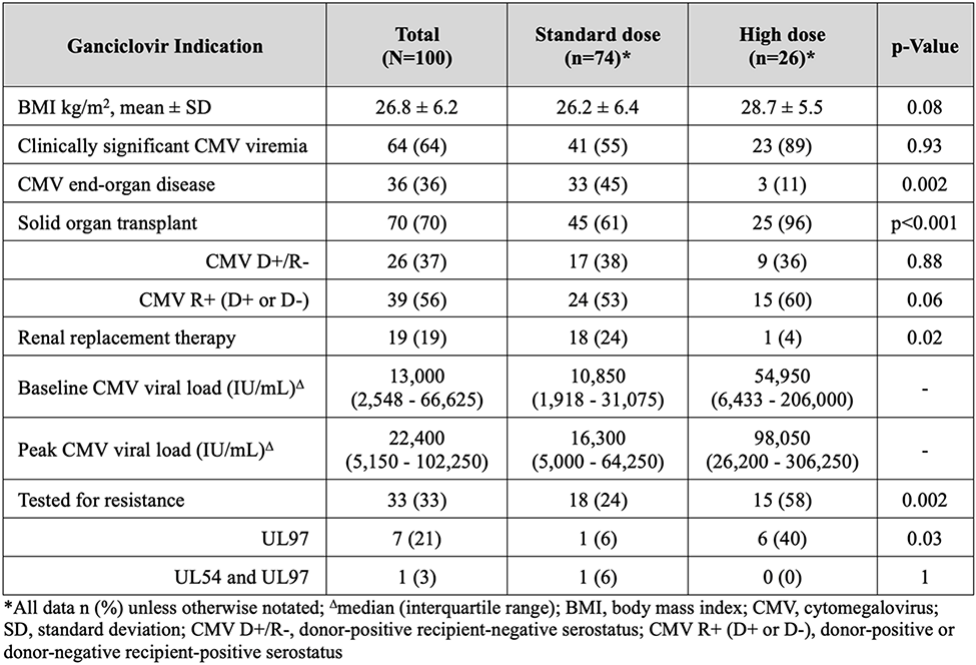
Baseline Characteristics

**Methods:**

This was a retrospective, multicenter cohort study of adults with CMV treated with ≥72 hours of GCV between May 2022 and August 2024. The primary outcome was the initial GCV regimen prescribed (Figure 1). Patients were categorized as primarily standard dose (PSD) or primarily high dose (PHD), defined by the regimen received for ≥50% of the GCV course. Clinical endpoints assessed were composite clinical failure and various safety measures (Figure 1). Additional secondary outcomes included rationale for HD GCV and indications for transition from SD to HD GCV.

**Figure 3:**
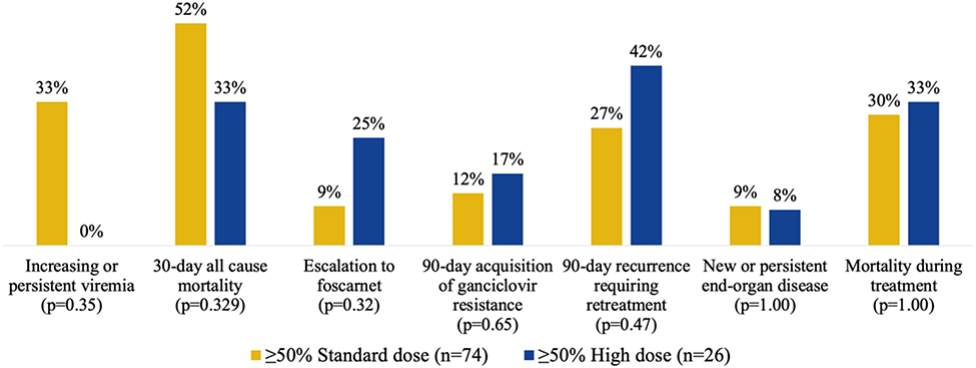
Reasons for Clinical Failure by Primary Dosing Regimen

**Figure 4:**
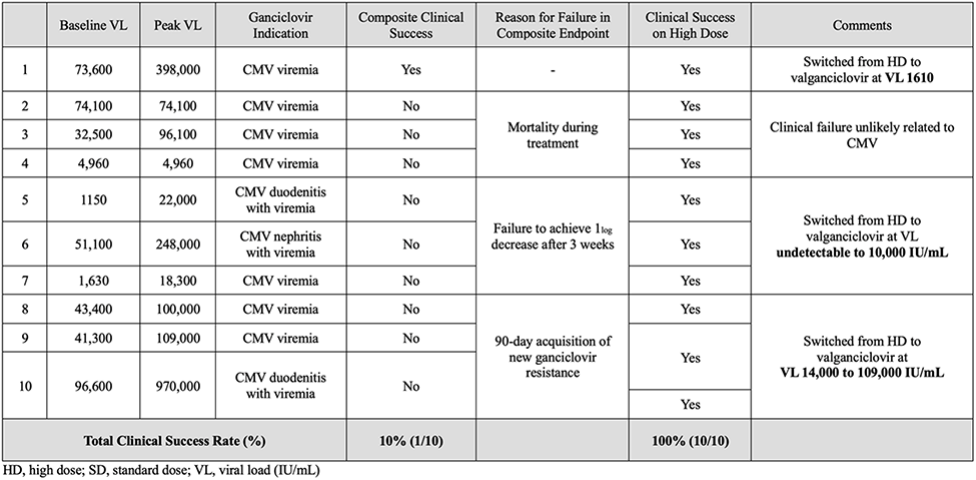
Patients Escalated from Standard to High Dose Ganciclovir due to Increasing or Persistent Viremia

**Results:**

One hundred patients (74 PSD vs. 26 PHD) were included with baseline characteristics shown in Figure 2. Initial regimens were 66% SD, 26% HD, and 8% alternative (below SD). The most common rationales for HD GCV were increasing or persistent viremia (IPV) (52%), provider preference (18%), and documented GCV resistance (14%). There was no significant difference in the rate of clinical failure (44.6% PSD vs. 46.2% PHD, p=0.89) or reasons for clinical failure between groups (Figure 3). Among the 10 patients who escalated from SD to HD GCV for IPV, 100% experienced clinical success while on HD, although 90% experienced clinical failure prior to the completion of treatment (Figure 4). New onset neutropenia (15% vs. 23%, p=0.34) and 90-day graft rejection (10% vs. 12%, p=0.72) were similar between groups.

**Conclusion:**

GCV dosing practices within our health system are provider-driven and heterogenous. Assessment of these practices is limited by retrospective chart review. No clear benefit of PHD was observed over PSD, nor were increased adverse effects noted in the PHD group. However, in patients changed from an SD to HD regimen, clinical success was observed on HD GCV, with subsequent failure after transition to an oral regimen. This suggests a need to further study optimal oral treatment strategies for patients with clinical success on HD GCV.

**Disclosures:**

All Authors: No reported disclosures

